# Lymphocytic Airway Inflammation in Lung Allografts

**DOI:** 10.3389/fimmu.2022.908693

**Published:** 2022-07-12

**Authors:** Jesse Santos, Daniel R. Calabrese, John R. Greenland

**Affiliations:** ^1^ Department of Medicine University of California, San Francisco, San Francisco, CA, United States; ^2^ Medical Service, Veterans Affairs Health Care System, San Francisco, CA, United States

**Keywords:** lung allograft, lung allograft immunity, lung allograft inflammation, NK cell, T cell, inflammation, lymphocyte

## Abstract

Lung transplant remains a key therapeutic option for patients with end stage lung disease but short- and long-term survival lag other solid organ transplants. Early ischemia-reperfusion injury in the form of primary graft dysfunction (PGD) and acute cellular rejection are risk factors for chronic lung allograft dysfunction (CLAD), a syndrome of airway and parenchymal fibrosis that is the major barrier to long term survival. An increasing body of research suggests lymphocytic airway inflammation plays a significant role in these important clinical syndromes. Cytotoxic T cells are observed in airway rejection, and transcriptional analysis of airways reveal common cytotoxic gene patterns across solid organ transplant rejection. Natural killer (NK) cells have also been implicated in the early allograft damage response to PGD, acute rejection, cytomegalovirus, and CLAD. This review will examine the roles of lymphocytic airway inflammation across the lifespan of the allograft, including: 1) The contribution of innate lymphocytes to PGD and the impact of PGD on the adaptive immune response. 2) Acute cellular rejection pathologies and the limitations in identifying airway inflammation by transbronchial biopsy. 3) Potentiators of airway inflammation and heterologous immunity, such as respiratory infections, aspiration, and the airway microbiome. 4) Airway contributions to CLAD pathogenesis, including epithelial to mesenchymal transition (EMT), club cell loss, and the evolution from constrictive bronchiolitis to parenchymal fibrosis. 5) Protective mechanisms of fibrosis involving regulatory T cells. In summary, this review will examine our current understanding of the complex interplay between the transplanted airway epithelium, lymphocytic airway infiltration, and rejection pathologies.

## Introduction

Since the first successful series of heart-lung and lung transplants in the 1980s, obliterative bronchiolitis has been recognized as the predominant pathologic finding of chronic lung allograft rejection. Both proliferative bronchiolitis, characterized by transluminal fibroproliferative tissue or Masson bodies, and constrictive bronchiolitis, characterized by concentric subepithelial fibrosis, were observed in these early allografts, typically surrounded by lymphocytes (1). Chronic lung allograft dysfunction (CLAD) is the syndrome of lung function decline in transplant recipients that is the major barrier to long term survival following lung transplant and includes both obstructive and restrictive phenotypes (2, 3). The obstructive phenotype is termed Bronchiolitis Obliterans Syndrome (BOS), because the predominant decline in one-second forced expiratory volume (FEV1) is presumed to be secondary to obliterative bronchiolitis pathology (3). Restrictive allograft syndrome (RAS) is pathologically associated with pleuro-parenchymal fibroelastosis (2). As was demonstrated in this original autopsy series, the pathologic hallmarks of BOS and RAS were frequently observed together (4, 5). Although, autopsy and explant studies typically reflect advanced or end stage lung disease which may limit conclusions drawn regarding disease processes marked by significant evolution. Further, advanced lung disease has significant tissue heterogeneity, rendering temporal conclusions involving focal or diffuse pathology challenging.

A similar syndrome of bronchiolitis obliterans is seen following allogeneic, but not autologous, stem cell transplant, suggesting that bronchiolitis obliterans results from an immune-mediated process. Indeed, increasing numbers of donor-recipient major histocompatibility complex (MHC) mismatches have been associated with risk of CLAD (6, 7). Even a minor histocompatibility antigen mismatch encoded by a single amino acid can drive obliterative airway disease, a murine analog of bronchiolitis obliterans, via CD8+ T cell-mediated alloimmune responses (8). In the absence of MHC mismatch between the lung and immune system, obliterative bronchiolitis is associated with some unusual exposures. Identified as a result of environmental exposures among popcorn factory workers, the butter flavoring butane-2,3-dione (diacetyl) covalently binds arginine residues in the small airways, forming haptens that trigger lymphocytic inflammation as a precursor to obliterative bronchiolitis (9–11). Together, these findings implicate lymphocytic immune responses in the airways as central to CLAD pathogenesis, as this review will explicate.

## Innate and Adaptive Lymphocytes in the Lung

Among transplanted solid organs, lung and intestine allografts have continual exposure to microbes and non-infectious environmental stimuli, necessitating mucosal-associated lymphoid tissue. Accordingly, lung allografts are predisposed to lymphocytic inflammation. The lung is notable for a diverse resident lymphocytic cell population at rest, and is a site for lymphocyte trafficking from peripheral reservoirs during acute injury (12). As such, across the various lung transplant inflammatory syndromes, lymphocytes can play a variety of roles. Where possible, this review attempts to distinguish disease processes where it is known that lymphocytes directly mediate injuries from those where there may be non-specific recruitment.

Innate lymphoid cells (ILCs) provide a first line of immunologic defense and are distinct from adaptive immune cells, discussed further below (**Table 1**). ILC activation is dependent upon integration of signals from cytokine stimulation, activating and inhibitory receptors, and physiological cues from their microenvironment (13, 14). There are three major ILC subsets: ILC1s defend against viruses and some bacteria primarily through cytokines like IFN-γ (interferon-gamma) and TNF-α (tumor necrosis factor-alpha). ILC2s classically respond to parasites and play important roles in allergic responses with cytokines like IL-4, IL-5, and IL-13 (18); and ILC3s play important antibacterial roles though IL-1β, IL-22, and IL-17. ILC1s and natural killer (NK) cells have overlapping roles and functions. Functionally, ILC1s are largely tissue-resident, while NK cells more commonly circulate and have greater cytotoxic function (12, 19). NK cells are a major source of IFN-γ in the lung and comprise up to 10% of the resident lymphocyte populations. NK cells mediate infectious and sterile lung diseases and have been implicated in both allograft injury and tolerance through a variety of mechanisms (12). NK cell function is determined by the integration of multiple activating and inhibiting signals from a variety of somatically-encoded receptors (20). As such, their role in directly mediating versus trafficking to sites of injury depends upon tissue contexts. For example, the role of NK cells during influenza infection is contested as some studies show NK depletion in experimental models leads to worse outcomes; whereas, other studies show no differences in experimental lung injury (21–23).

**Table 1:** Overview of airway lymphocyte types.

Lymphocyte types	Key subtypes	Activation signals	References
**Innate lymphoid cells**	ILC1, ILC2, ILC3	Cytokines	(13, 14)
**NK cells**	Cytotoxic, Cytokine secreting	Missing self, stress molecules, antibodies	(12)
**T cells**	Cytotoxic, Helper (Th1, Th2, Th17), Regulatory, Follicular helper	Intracellular or extracellular peptides presented on MHC to T cell receptors (CD3).	(15)
**B cells**	Naïve B cells, germinal center B cells differentiate into plasma cells	Extracellular antigens binding to B cell receptor.	(16, 17)

T cells develop in the thymus, where T cell receptor (TCR) genes rearrange to generate a diverse array of receptors that are subsequently selected for low-level binding to self-antigens. Recognition of near-self antigens makes T cells adept at recognizing virally infected cells, but also explains how auto- and alloimmune responses develop. In fact, 5-15% of circulating T cells will typically react to donor alloantigen, depending on HLA (human leukocyte antigen) mismatching and recipient immune status (24, 25). T cells are further subdivided based on function and cellular markers into 3 major groups: CD4+ T cells, CD8+ T cells, and γδ T cells. Helper CD4+ T cells primarily secrete cytokines to drive immune response and provide co-stimulation to drive cytotoxic CD8+ T cell and B cell humoral responses (26, 27). The types of cytokines produced by helper T cells lends to their subcategorization into Th1, Th2, Th17, and T regulatory subsets. There is some debate in the literature over the relative contributions of helper T subtypes, but there is evidence supporting a role for all four (15, 28, 29). Th1, Th2, and Th17 phenotypes are analogous to ILC1, ILC2, and ILC3 subclasses and are mediated by similar transcription factors, Tbet, GATA3, and RORγT, respectively (30–32). Like NK cells, CD8+ T cells have cytotoxic properties and secrete perforin and granzymes to lyse virally infected or malignant cells. Within this construct of innate and adaptive lymphoid cells lies a multitude of pathways to mediate injury, either non-specifically or in a targeted fashion. Following transplantation, donor antigens can be presented on either donor or host antigen presenting cells, resulting in direct or indirect antigen presentation, respectively (33).

B cells and plasma cells comprise the final major category of lymphoid cells and are responsible for producing antibodies. As with T cell maturation, B cell diversity is determined by somatic recombination, although B cells undergo a subsequent optimization step, called somatic hypermutation to heighten antigen specificity. B cells are activated by APCs (antigen presenting cell) and CD4+ T cells and contribute to acute and chronic allograft dysfunction through the process of antibody mediated rejection (AMR) (16, 17). As such B cells and plasma cells mediate allograft injury by directing effector cells to tissue deemed “non-self.”

While most lymphocyte populations amplify the cascade of responses that promote inflammation, there are a collection of T cell and B cell subsets which work to dampen this process. Regulatory immune cells help to limit the amount of collateral damage from the innate and adaptive immune systems. Regulatory T cells (Tregs) impair the expansion of conventional T lymphocytes, dampen T cell function, secrete immunosuppressive cytokines, adsorb proinflammatory cytokines, potentiate tolerogenic APCs, and create an environment to facilitate expansion of other Tregs (34). Preclinical studies in mouse models of solid organ transplant, have shown long-term graft acceptance by augmentation of Treg populations in transplant recipients (35, 36). Regulatory B cells (Bregs) are proposed to play a key role in homeostasis after lung transplant (37–39). Breg features may contribute to tolerance, making it possible to reduce immunosuppression (40). Finally, while NK cells do not have a specific regulatory subset, their actions may be curtailed via inhibitory surface receptor signaling. NK cells may also perform regulatory functions such as targeting pro-inflammatory cells, in certain contexts (41).

## Lymphocytic Inflammation in the Context of Primary Graft Dysfunction (PGD)

Primary graft dysfunction (PGD) is a syndrome of acute lung dysfunction in the early transplant period. Clinically, it is defined as multi-lobar chest X-ray opacifications and a decreased ratio of arterial oxygen to inspired oxygen (PaO2/FiO2) within the first 72 hours post-transplant. PGD is graded from absent (grade 0) to severe (grade 3). Severe PGD accounts for 30% of mortality in the first 30 days after transplant, 50% of the mortality within the first year of transplant and has been associated with lower baseline lung function and risk of CLAD (42, 43). PGD is the clinical manifestation of the pathologic process of ischemia-reperfusion injury (IRI) (44). Accordingly, PGD risk is dependent on the severity of ischemic injury, including warm and cold ischemic time. Allograft ischemia is further potentiated by chronic hypoperfusion, as bronchial arteries are not typically re-anastomosed during transplant. Advancements in surgical technique and allograft handling have reduced rates and severity of ischemia through limited use of cardiopulmonary bypass, limiting intra-operative blood transfusions, and limiting fraction of inspired oxygen intraoperatively (45–49). PGD risk is also driven by non-ischemic mediators of graft injury, including recipient BMI, donor tobacco use, and operative transfusions as stated above (47). Such factors may contribute to PGD by potentiating inflammation.

**Figure 1:**
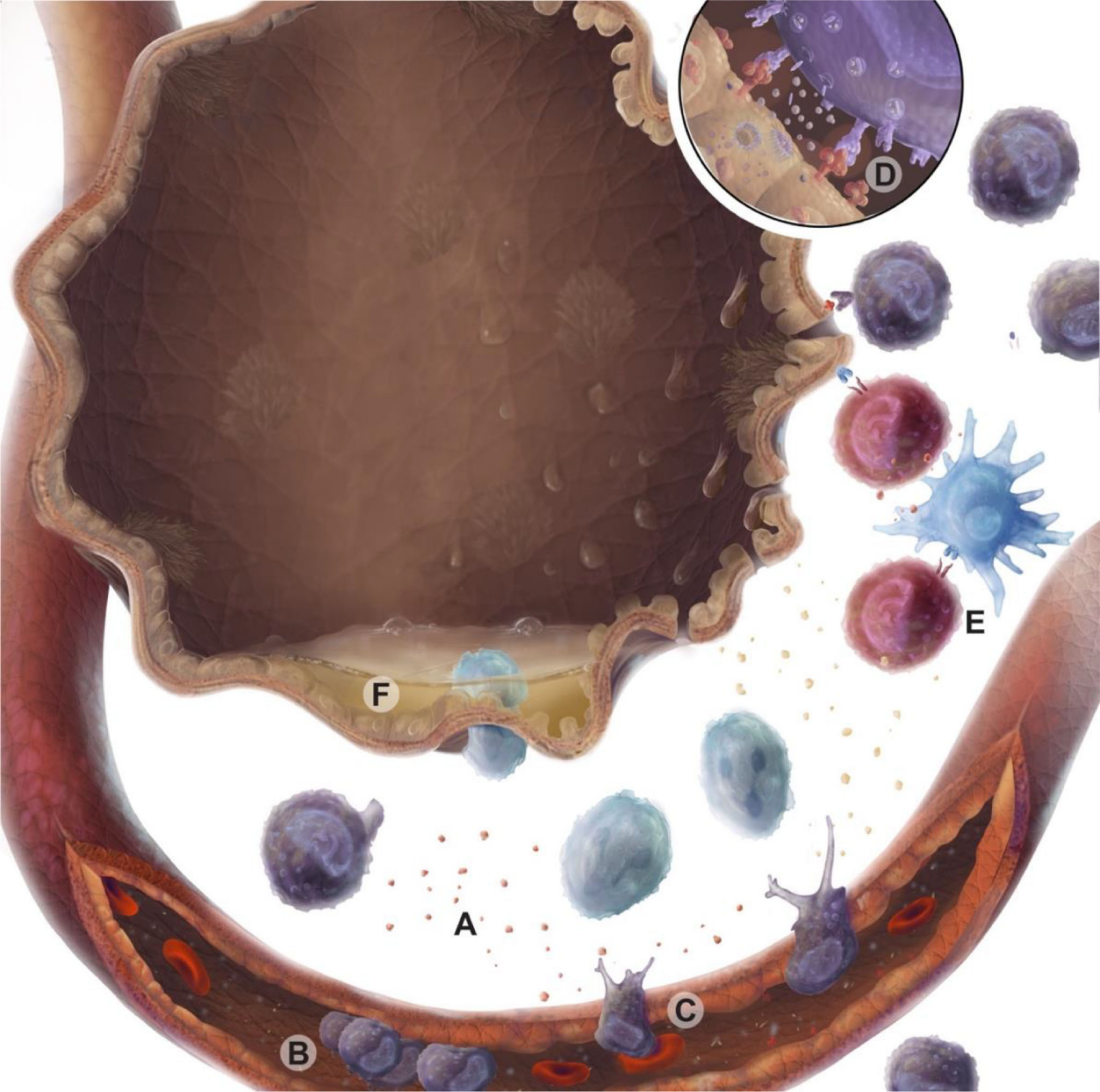
Immune cell responses during ischemia reperfusion injury (IRI). Warm ischemia, cold ischemia, and subsequent reperfusion with oxygenated blood lead to oxidative and mitochondrial cell stress, which are associated with epithelial injury. These injured epithelial cells produce damage molecular patterns (DAMPs) and chemokines **(A)** that recruit and activate immune cells *via* the vascular endothelium. Activated endothelium tether passing leukocytes from the circulation *via* selectins and integrins, causing immune cells to roll and adhere **(B)** prior to transmigration across a chemotactic gradient **(C)**. Lymphocyte activation is driven through MHC binding to T cell receptors or NK cell receptor ligand interactions. These activated lymphocytes may secrete cytotoxic perforin and granzyme molecules **(D)**. Professional antigen presenting cells can also present alloantigen to T cells amplifying graft-specific responses in response to injury **(E)**. Epithelial cell loss of tight junctions and breakdown results in barrier dysfunction and interstitial edema **(F)**.

IRI is primarily mediated by the innate immune system but can be further amplified through adaptive immune responses (**Figure 1**). Experimental and clinical data suggest a biphasic nature to this inflammatory process. The early phase of IRI is marked by oxidative stress, epithelial and endothelial dysfunction leading to further injury. Airway epithelial cells release chemokines and damage-associated molecular patterns (DAMPs) (50, 51), while endothelial cells upregulate adhesion markers (50, 52). Within murine models, oxidative stress measured via isoprostanes, was increased after IRI and could be mitigated by administration of azithromycin (53), These signals recruit and activate innate immune cells, including neutrophils and macrophages, and drive antigen presentation (54, 55). Accordingly, macrophage depletion is associated with reduced lung injury in murine models of PGD (56, 57). IL-17 and DAMPs promote neutrophil migration to the interstitial space. Neutrophils can amplify IRI through neutrophil extracellular traps (58, 59). CD1d-restricted NKT cells (natural killer T cell) have been shown to secrete IFN-γ; and help recruitment of neutrophils to the site of injury, suggesting innate immune cells may play an important role as a major source of IFN-γ in the lung (60).

Innate and adaptive lymphocytes play a key role in bridging early and late IRI. In both mouse models of IRI and in human lung transplant recipients following PGD, NK cells are observed in and around airways (61). By contrast, in lung allograft biopsies taken peripherally (excluding airways) before implantation and immediately after reperfusion NK cell populations are decreased (62). This would suggest the airways as central sites of NK-cell mediated IRI. The NKG2D receptor on NK cells recognizes stress molecules that are absent or lowly expressed at baseline but rapidly increased in response to a variety of injurious stimuli (63). In mouse models of IRI, NKG2D receptor stress ligands were shown to be increased on pulmonary endothelial and epithelial cells (61). Further, blockade of the NKG2D receptor or genetic deletion of the receptor on NK cells alone, was enough to abrogate pulmonary injury in these mouse models. Although, it should be repeated that NK cells predominantly influence the early phase of IRI, with other cell populations becoming more important as the initial wave of injury subsides. Consequently, renal models of IRI also show a similar role for NK cells in mediating renal tubule epithelial cell injury. This suggests that NK cells, from the moment of reperfusion, may be critical in translating epithelial cell stress during IRI to allograft damage.

These early reperfusion responses of NK cells may potentiate long-term outcomes by killing graft APCs. NK cell activity against APCs occurs in the setting of licensing mismatch (61). During NK cell development, NK cells express inhibitory receptors to host MHCI molecules to avoid self-cytotoxicity. During transplant with a mismatch in donor and recipient MHCI, this inhibitory signal is absent which releases NK cells for activation. For example, NK cells from an HLA-Bw4 positive recipient are licensed to Bw4 antigen and will kill APC lacking Bw4 antigens. This phenomenon plays a critical role in allogenic stem cell transplant, where graft versus host NK activity can prevent leukemia relapse (64). In a mouse skin transplant model of NK licensing mismatch, graft-derived APCs were largely destroyed by donor NK cells and skin allograft survival was improved via reduced antigen presentation to recipient lymphocytes (41, 65). A similar phenomenon has been observed in mouse lung transplant models, where NK cells could improve tolerance of an orthotopic lung allograft in a perforin dependent manner and in association with dendritic cell depletion (66). While this is predominantly animal model evidence there is some data pointing to HLA Bw4 mismatching that potentiates NK cell host-versus-graft activity has been linked to improved outcomes in two cohorts of lung transplant recipients (41).

Conventional lymphocytes are also implicated in driving the lung injury of IRI (67). IRI may potentiate HLA- or neo-antigen presentation and subsequent alloimmune responses (68, 69). While there is not a prominent influx of CD4+ T cells into the allograft during experimental IRI, depletion of CD4+ T cells attenuates injury. This suggests that CD4+ T cells have other roles than direct injury, such as recruitment of effector cells (70). Although, this also points towards CD4+ T cells being complimentary to other underlying disease processes. Within severe combined immunodeficient (SCID) mice a documented lack of lymphocytes caused decreased neutrophil invasion into ischemic lungs (71). A deeper look into this process shows that lymphocyte attraction of neutrophils occurs as early as during warm ischemia time (72, 73). Finally, IRI may also amplify anti-donor anti-MHC and anti-autoantigen antibody production (61).

## Acute Cellular Rejection Pathologies and the Significance of Airway Inflammation

Acute lung allograft rejection is mediated via two primary pathologies: acute cellular rejection and antibody mediated rejection. Some degree of acute cellular rejection (ACR) occurs in up to 30% of all lung transplants within the first post-operative year (74). ACR is predominantly a T cell mediated process. Recipient-derived effector memory T cells infiltrate the allograft traversing vascular endothelium, proliferate and migrate to the airways, where they can persist as resident memory cells (75). The diagnosis of ACR is currently confirmed with transbronchial biopsies and quantified based on standardized histopathologic patterns (76, 77). Risk factors for ACR include the degree of human leukocyte antigen mismatching and genetically determined differences within the innate and adaptive immunologic responses of the recipient (78–80). A-grade rejection refers to a mononuclear perivascular infiltrate. B-grade rejection refers to lymphocytic bronchitis or small airway inflammation. Lymphocytic bronchiolitis after transplant is linked to worse CLAD-free survival (81). C-grade rejection refers to obliterative bronchiolitis on transbronchial biopsy. However, this finding is neither sensitive nor specific for CLAD. D-grade rejection denotes accelerated graft atherosclerosis, which is not typically seen on transbronchial biopsies. Finally, E-grade rejection is not a part of standard ISHLT criteria but refers to lymphocytic inflammation on endobronchial (large airway) biopsies (82).

While B-grade rejection is generally assessed on transbronchial biopsies, similar criteria can be used to grade airway inflammation on large airway endobronchial biopsies. In a single center study, diagnosis of E-grade rejection within the first year after transplant was associated with a subsequent 1.8-fold increased risk of CLAD or death. Interestingly, gene expression profiling of A-, B-, and E-grade rejection pathologies identified signatures of allograft rejection that are shared across solid organ transplant, suggesting that these histopathologic findings may share a common pathobiology (82).

Much of the effect seen in E-grade rejection was attributable to high-grade lymphocytic bronchitis (83). The presence of lymphocytic inflammation on transbronchial or endobronchial biopsies has been termed Lymphocytic Airway Disease (LAD). In a separate study, LAD was associated with a 1.6-fold increased risk of CLAD or death. Interestingly, this association was limited to the cohort not taking azithromycin for CLAD prophylaxis (84). The use of azithromycin has been suggested to improved lung function after development of BOS as well as improve overall survival, when used as rescue therapy (85–87). There is evidence in animal models that azithromycin may be linked to reduced production of IL-17 from Th17 cells (88). At our center, we observed a decreased incidence of lymphocytic bronchitis since the introduction of azithromycin for CLAD prophylaxis (89). However, data are mixed regarding the effectiveness of azithromycin on improving CLAD-free survival or overall survival when used prophylactically (90–92). Additionally, the mechanism whereby azithromycin reduces airway inflammation remains unclear. However, there is some evidence supporting multiple pathways via; the reduction in free radicals, suppression of vascular endothelial growth factor’s (VEGF) effects on angiogenesis, and the reduction of gastroesophageal reflux owning to azithromycin’s gut motility effects (53, 93, 94).

Young age is also associated with a higher rate of acute rejection within the first year after transplantation, perhaps owning to a stronger immune response or exposure to a diverse antigens as recipients age lends to less immunogenic responses (95). ACR in the pulmonary allograft is a serious complication that is both an acute cause of graft-dysfunction and inflammation-related morbidity, but also a major risk factor for the development of CLAD (96). Acute rejection contributes to some low risk of mortality, particularly in the first year after lung transplantation, representing approximately 3.3% of all deaths within the first 30 days (95).

Antibody-mediated rejection is rarer in the context of lung transplantation and occurs when de novo or pre-formed antibodies against donor antigens trigger cell injury via two primary pathways. In the classic pathway, complement-binding antibodies activate the complement cascade resulting in membrane attack complex formation and direct target cell death. However, injury may also occur when antibodies bound to target are non-specifically recognized by cells carrying Fc-receptors leading to a process termed antibody-dependent cell mediated cytotoxicity (ADCC). Irrespective of mechanism, the increased frequency of de novo donor-specific antibodies (DSA) is associated with increased risk of CLAD (97, 98). While antibodies against donor antigens are common and associated with CLAD, definitive acute AMR occurs in fewer than 5% of all lung transplant recipients (99, 100). The development of DSA depends on T follicular helper cell interactions with B cells, including CD28-dependent co-stimulation (101). Thus, DSA may be a marker for alloimmune activation as much as biological mediator. Neutrophils, macrophages, and NK cells have been implicated in ADCC. Though, NK cells are thought to be the primary effector cell in human ADCC as their Fc receptor, CD16, is activating-only. In contrast, CD32 and CD64 lead to a mix of activating and inhibiting signals. In support of this mechanism, CD16 polymorphisms that enhance ADCC are associated with increased CLAD risk (102, 103). Thus, the roles of lymphocytes and airway inflammation in AMR require further investigation.

There are two pathways of allorecognition implicated within ACR, the direct and indirect pathways. In the direct pathway, donor APCs migrate to secondary lymphoid tissue and present alloantigen directly to recipient T cells. In the indirect pathway, recipient APCs present alloantigen derived from dying donor APCs to T cells, either in the secondary lymphoid organs or in the allograft itself (104). ACR is suspected to reflect the direct pathway (105), and ACR is associated with increased in donor-specific CD8+, conventional CD4+, and regulatory T cell responses in the peripheral blood (24). Within other solid organ transplant models, recipients one year post-transplantation may demonstrate hypo-responsiveness to alloantigen via the direct pathway (105–107). This type of partial tolerance to donor MHC is inconsistently observed following lung transplantation and may depend on conventional or regulatory T cell immune senescence (108). Conversely, one year post-transplantation, recipients show hyper-responsiveness towards alloantigen via the indirect pathway, where “primed” T cells have been identified on bronchoalveolar lavage (BAL) (106, 107). Thus, repeated rejection could lead to CLAD via either pathway.

ACR has important limitations as a predictor of CLAD development. While both A- and B-grade rejection have been linked to CLAD (81), the association between A1-grade ACR and CLAD risk is inconsistent (83, 109, 110). This perhaps points to a common theme among several studies that although in the acute setting lymphocytic inflammation is a major contributor of injury long term outcomes likely are underpinned by a multitude of inflammatory mediators and effects.

ACR is typically heterogenous and sometimes a symptomatically silent process. There is poor interobserver reliability for ACR grading across sites, with a Cohen’s kappa value of 0.18 to 0.48 for A-grade rejection and 0.04 to 0.47 for B-grade (111, 112). Inadequate tissue sampling is an issue for both grades, but insufficient airway tissue for confident assessment of B-grade rejection has been reported in up to two-thirds of transbronchial biopsies (113). During incipient CLAD, with active decline in FEV1, there are no reliable histopathologic correlates on transbronchial biopsy (113). ACR diagnosis can depend upon institutional surveillance and biopsy protocols. Multiple studies have identified gene expression or BAL cell counts or cytology as better predictors of CLAD than ACR itself (114–117). For example, a gene signature of lymphocytic bronchitis assessed in small airway cytologic brushings identified cases of FEV1 decline that would go on to death or retransplant in the next two years, even when transbronchial biopsies showed no evidence of rejection (113). These inconsistencies suggest that these sampling and interpretation issues may be under appreciated on transbronchial biopsy and have led to an underappreciation of the importance of airway inflammation leading to CLAD. Gene expression-based diagnostics using BAL or airway brushes would sample a larger proportion of small airway tissue, may facilitate detection of airway inflammation, and could guide potential therapies to reduce CLAD progression (29, 115).

## Potentiators of Lymphocytic Airway Inflammation and Heterologous Immunity

**Figure 2:**
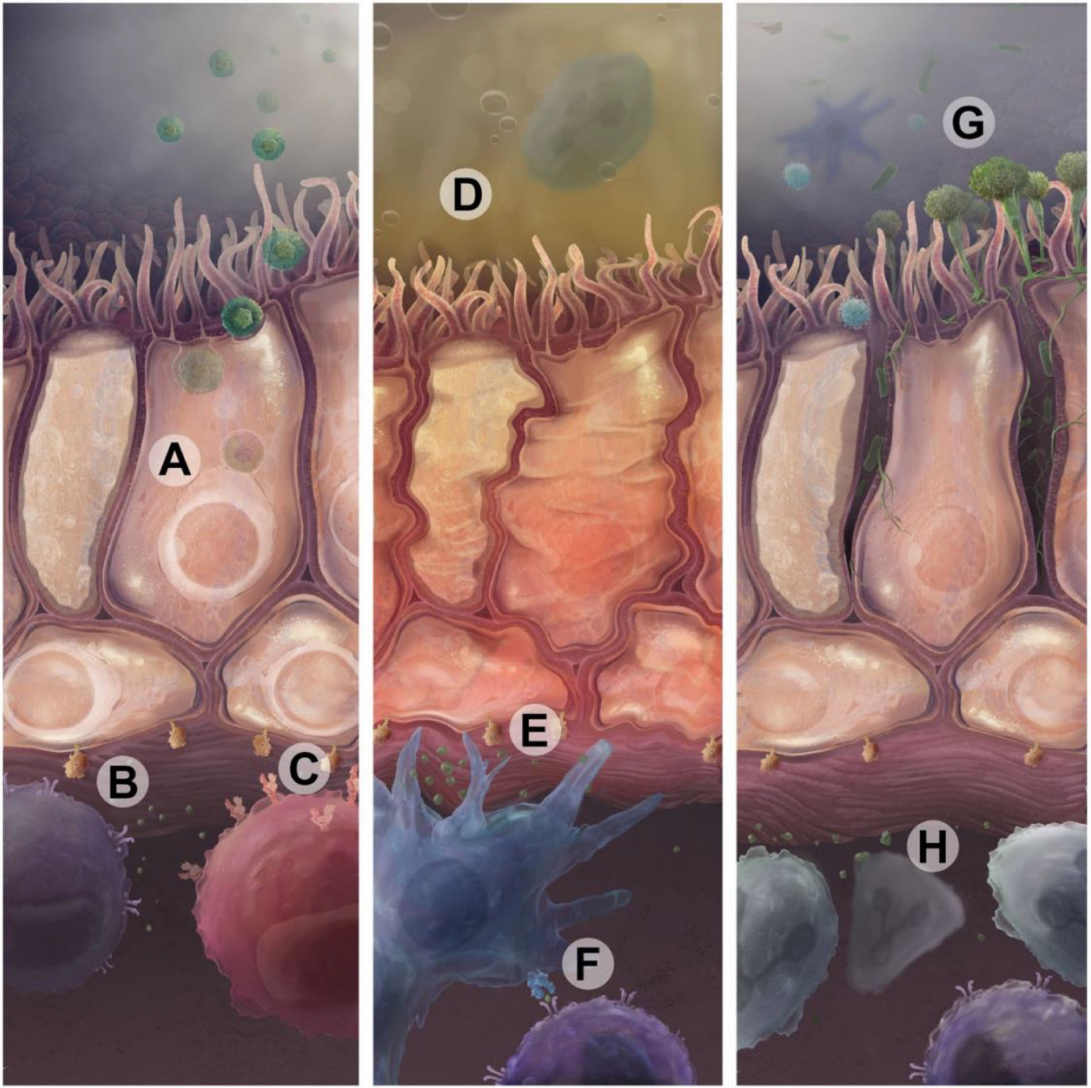
Infectious and non-infectious insults drive immune activation that can lead to CLAD. **(A)** CMV or other respiratory viral infections in epithelial cells augment antigen presentation through upregulation of donor-derived MHC and β2-microglobuilin, shown in **(B)**. These MHC complexes present viral antigens and participate in direct presentation of donor antigens to T cell receptors. CMV antigens are also presented on HLA-E to activating NKG2C receptors on NK cells **(C)**. Sterile injury, such as through exposure to gastric acid reflux **(D)** or air pollution, can cause direct airway cell injury which also leads to upregulation of antigen presentation and proinflammatory cytokines **(E)**. Recipient antigen presenting cells then present alloantigens through the indirect pathway using recipient MCH or through the semi-direct pathway using acquired donor MHC molecules **(F)**. This can drive lymphocytic immune responses specific to donor antigens or unmasked self-antigens. **(G)** Bacterial and fungal infections can serve as an acute or persistent source of pathogen-associated molecular patterns that drive immune responses in lymphoid and myeloid immune cells **(H)** *via* Toll-like receptors, Dectin-1, or other pathways.

Airway inflammation may be challenging to quantify but can yield insights into alloimmune responses and the risk for progression to CLAD. However, there are multiple drivers of airway inflammation outside of alloimmune responses that are relevant to long term lung transplant outcomes including air pollution, infections, and aspiration of gastric acid (**Figure 2**) (118).

Lung transplant recipient exposure to air pollution, as quantified by the concentration of particulate matter less than 10 micrometers in diameter (PM10), is associated with increased risk of airway inflammation on biopsy and in BAL in the 2–3 days following exposure (84). In a study including 13 centers in Europe, PM10 and proximity to roads were associated with worse CLAD-free survival (119). Interestingly, azithromycin appeared to mitigate this effect.

Infections may stimulate alloimmune responses and precipitate CLAD development directly and through increased ACR (120). Bacterial infections like Pseudomonas, as well as infections from fungi like Aspergillus may affect CLAD risk through impacts on inflammation, airway epithelial cells, and other constituents of the respiratory microbiome (121, 122). Lung transplant recipients are at particular risk for community-acquired respiratory virus (CARV) infections: respiratory syncytial virus (RSV), coronavirus, rhinovirus, influenza, and parainfluenza viruses (123). Several studies independently demonstrate that community respiratory virus infections convey an increased risk of CLAD development. When stratified between upper and lower viral respiratory tract infections there is an increased risk, almost 3-fold, for lower respiratory tract viral infections (124). Additionally, there appears to be a temporal component to the development of CLAD and onset of respiratory viral infection (RVI), where a recent infection confers a larger risk of CLAD development (125). CARV infection within the first year of transplant confers a risk to CLAD development several years thereafter (126). Early treatment of RSV infection decreased the incidence of new or progressive CLAD (127).

CARV infection may drive airway inflammation and subsequent CLAD through multiple mechanisms. In a rat model of lung transplantation, parainfluenza virus infection potentiated lymphocytic inflammation and obliterative airway disease in allogeneic lungs relative to syngeneic or uninfected lungs (128). Viruses are potent inducers of interferons and interferon-associated chemokines can recruit cytotoxic lymphocytes to airways. Specifically, in CARV-infected lung transplant recipients, higher concentrations of chemokine C-X-C motif ligand 10 (CXCL10) and C-C motif chemokine ligand 11 (CCL11) predicted FEV1 decline over the next 6 months (129). CARV infection can impair regulatory T cells and expose cryptic antigens leading to de novo anti-ColV and k-alpha1 tubulin antibodies that are associated with CLAD (130). Viral infections can also lead to the release of exosomes containing self-antigens that can trigger responses to self-antigens and CLAD pathology (131). Viral infections can potentiate donor-specific immune responses through heterologous immunity. For example, CD8+ T cells specific for Human cytomegalovirus (CMV) or Epstein-Barr virus (EBV) have been shown to cross react with donor alloantigen (132). NK cells can also mediate recall immune responses to CMV through the NKG2C receptor, and elevations in NKG2C+ NK cells in the BAL is a risk factor for CLAD (63).

CMV infection, within immunocompetent hosts, establishes immunity which controls infection even if the virus is reactivated (133). However, there is evidence to suggest CMV infection may cause life-threatening complications in organ transplant recipients and has been associated with more frequent acute and chronic rejection (134–136). CMV-reactive T cells can cause tissue damage by several mechanisms: (i) direct cytotoxic effect on CMV infected (allograft) cells, (ii) indirect bystander activation and proinflammatory milieu formation, and (iii) heterologous (cross-reactive) allorecognition (137). The cross-reactivity of CMV-reactive effector T cells to HLA class I antigens is widely accepted and have been isolated from the peripheral blood of kidney transplant recipients (132, 138, 139).

Chronic exposure to gastric acid secondary gastroesophageal reflux disease (GERD) has also been shown to be associated with the development of CLAD (140). Gastric acid may directly trigger lymphocytic airway inflammation. For example, chronic exposure to gastric fluid in rodent lung transplant models is associated with ACR, peribronchial T cell infiltration, T cell-dependent cytokine release in BAL, and increased frequencies of obliterative bronchiolitis lesions (141, 142).Conversely, anti-reflux surgery is associated with decreased BAL lymphocytes and neutrophils (143). For these reasons, many centers will perform anti-reflux surgery for lung transplant recipients with uncontrolled GERD and risk of CLAD progression (143–145).

## Airway Inflammation in the Pathogenesis of CLAD

CLAD pathology may reflect a final common pathway of injury responses leading to airway remodeling and fibrosis. For example, neutrophils in BAL fluid are identified as a reversible CLAD risk factor. A syndrome >15% BAL neutrophils and ≥10% decreased in FEV1 that reverses with azithromycin treatment is termed azithromycin-responsive allograft dysfunction (ARAD), previously known as neutrophilic reversible allograft dysfunction (NRAD) (146). ARAD is closely linked with lymphocytic airway inflammation and may reflect a paradoxical IL-17-dependent production of IL-8 in airway epithelial cells exposed to tacrolimus that is reversed by azithromycin (87, 147). Nonetheless, while azithromycin prophylaxis can potently reduce airway inflammation, it has been inconsistently associated with CLAD prevention (91, 148). That lung transplant recipients continue to develop CLAD despite azithromycin prophylaxis suggests multiple pathways to CLAD.

Airway inflammation can induce and activate myofibroblasts. These cells deposit the extracellular proteins like collagen and fibronectin that constitute airway fibrosis (149). Myofibroblasts may derive from airway epithelial cells via epithelial to mesenchymal transition (EMT) as well as from pericytes via pericyte-mesenchymal transition (PMT) (150–152). Pathologic EMT can be triggered by lymphocyte activation and secretion of transforming growth factor-beta (TGF-β). Mouse models with knockout of TGF-β show protection from fibrosis and EMT (153–155). Growth factors such as VEGF and TGF-β also mediate interactions between the lung endothelium and pericytes and have been independently studied as drivers of fibrosis (156, 157). Myofibroblasts can also differentiate from donor-derived resident mesenchymal stem cells in response to Th2 lymphocytic inflammation (158).

Club cell dysfunction can also drive CLAD pathology. Club cells are non-ciliated epithelial cells typically found within bronchioles that promote injury repair and secrete anti-inflammatory proteins (159–162). Club cell depletion leads to CLAD-like pathology that can be prevented with CD8+ T cell depletion (163). Club cells can proliferate rapidly and differentiate into airway epithelial cell populations. Such proliferation puts stress on cell replication machinery, including telomeres, the nucleoprotein caps on chromosomes. Telomere dysfunction in the allograft has been associated with CLAD risk and induction of club cell telomere dysfunction in mice drives both lymphocytic airway inflammation and CLAD-like pathology (164, 165).

## Conclusions

CLAD is primarily a disease of airway or parenchymal fibrosis resulting from alloimmune responses and lymphocytic airway inflammation is likely to be a major driver of CLAD pathology. However, lymphocytic airway inflammation can be challenging to detect using standard of care histopathologic analysis on transbronchial biopsies. Transcriptional analysis of airway brushings biopsies, or BAL fluid may allow more reliably detection of pathogenic airway inflammation (29, 113, 121). Airway lymphocytes include ILCs, T cells, B cells, and NK cells, which have distinct roles in PGD, ACR, AMR, and CLAD. Together with acute peri-vascular rejection, antibody-mediated responses, ischemia-reperfusion injury, graft infections, and gastroesophageal reflux disease, airway inflammation appears to drive an inflammatory milieu leading to airway-centric fibrosis (6, 24, 81, 83, 116, 166–169).

At the same time there are some limitations to the current data linking airway lymphocytes to rejection pathology. The observation of lymphocytes coincident with graft pathology does not imply lymphocytes are causal. These lymphocytes could be a consequence of injury, or actively counteracting pathology, such as with regulatory T and B cells (24). While there are some causal data from rodent lung transplant models, the models have limitations and may not always match human immunobiology (170). Additionally, lymphocytes are only a component of the immune cells contributing to lung injury, as neutrophils, monocytes, and other cells also play key roles.

Targeting immune suppression to airway lymphocytes is a promising strategy to prevent or delay CLAD. For example, a trial of inhaled cyclosporin showed encouraging results, even though it was terminated early for business reasons (171). The JAK-1 inhibitor itacitinib has shown promise as inhibitor of lymphocytic mucosal inflammation and is under investigation to address inflammation in the context of early CLAD (172). The use of azithromycin as prophylaxis for CLAD or as a rescue from BOS has been implemented by several institutions, as detailed previously with varying degrees of success (85–87, 90, 92). Also, an adenosine A2A receptor antagonist is under investigation to reduce invariant NKT cell mediated inflammation in PGD (173). Other strategies to dampen airway inflammation, such as regulatory T cell adoptive therapy and/or pretransplant allograft modification during ex vivo lung perfusion, have shown preclinical promise as adjuncts to traditional immune suppression (174). A fair portion of our understanding of allograft injury comes from in vitro, ex vivo, and animal models which are extremely important in studying the biology that informs our clinical pursuits. However, it is vital to continue to test these theories within robust and safe clinical trials.

## Author Contributions

All authors contributed to manuscript revision, read, and approved the submitted version.

## Conflict of Interest

The authors declare that the research was conducted in the absence of any commercial or financial relationships that could be construed as a potential conflict of interest.

## Publisher’s Note

All claims expressed in this article are solely those of the authors and do not necessarily represent those of their affiliated organizations, or those of the publisher, the editors and the reviewers. Any product that may be evaluated in this article, or claim that may be made by its manufacturer, is not guaranteed or endorsed by the publisher.

## Glossary

**Table:**

ACR	acute cellular rejection
ADCC	antibody-dependent cell-mediated cytotoxicity
AMR	antibody mediated rejection
APC	antigen presenting cell
ARAD	Azithromycin-responsive allograft dysfunction
BAL	bronchiolar lavage
BOS	bronchiolitis obliterans syndrome
Breg	regulatory B cell
CARV	community-acquired respiratory virus
CCL11	C-C motif chemokine ligand 11
CD	cluster of differentiation
CLAD	chronic lung allograft dysfunction
CMV	human cytomegalovirus
CXCL10	chemokine C-X-C motif ligand 10
DAMP	damage-associated molecular pattern
DSA	donor-specific antibody
EBV	Epstein-Barr virus
EMT	epithelial mesenchymal transition
FEV1	one-second forced expiratory
GERD	gastroesophageal reflux disease
HLA	human leukocyte antigen
IFN-γ	interferon-γ
IL	interleukin
ILC	innate lymphoid cell
IRI	ischemic reperfusion injury
AK-1	Janus kinase 1
LAD	lymphocytic airway disease
MHC	major histocompatibility complex
NK	natural killer
NKT	natural killer T cell
NRAD	neutrophile reversible allograft dysfunction
PaO2/FiO2	arterial oxygen partial pressure to fraction of inspired oxygen ratio
PGD	primary graft dysfunction
PM10	Particulate matter under 10 micros in size
RAS	restricted allograft syndrome
RORγT	retinoic acid-related orphan receptor-γ
T RSV	respiratory syncytial virus
RVI	respiratory viral infection
SCID	severe combined immunodeficiency
Tbet	T-box expressed in T cells
TCR	T cell receptor
TGF-β	transforming growth factor-β
TNF-α	tumor necrosis factor-α
Treg	regulatory T cell
